# New RO TFC Membranes by Interfacial Polymerization in *n*-Dodecane with Various co-Solvents

**DOI:** 10.3390/membranes6020024

**Published:** 2016-04-29

**Authors:** Abdullah Sulaiman Al-Hobaib, Mohammed Sulaiman Al-Suhybani, Khalid Mohammed Al-Sheetan, Hasan Mousa, Mohammed Rafi Shaik

**Affiliations:** 1 Nuclear Science Research Institute, King Abdulaziz City for Science and Technology (KACST), P.O. Box 6086, Riyadh 11442, Saudi Arabia; sohybani@kacst.edu.sa (M.S.A.-S.); ksheetan@kacst.edu.sa (K.M.A.-S.); 2 Department of Petroleum and Chemical Engineering, Sultan Qaboos University, P.O. Box 33, Muscat 123, Oman; 3 Department of Chemical Engineering, Jordan University of Science and Technology, P.O. Box 3030, Irbid 22110, Jordan; 4 Department of Chemistry, College of Sciences, King Saud University (KSU), P.O. Box 2455, Riyadh 11451, Saudi Arabia

**Keywords:** interfacial polymerization, RO membrane, co-solvent, desalination

## Abstract

The objective of this research is to prepare and characterize a new and highly efficient polyamide TFC RO membrane by interfacial polymerization in dodecane solvent mixed with co-solvents. Three co-solvents were tested namely; acetone, ethyl acetate, and diethyl ether of concentration of 0.5, 1, 2, 3, and 5 wt %. The modified membranes were characterized by SEM, EDX, AFM and contact angle techniques. The results showed that addition of co-solvent results in a decrease in the roughness, pore size and thickness of the produced membranes. However, as the concentration of the co-solvent increases the pore size of the membranes gets larger. Among the three co-solvents tested, acetone was found to result in membranes with the largest pore size and contact angle followed by diethyl ether then ethyl acetate. Measured contact angle increases as the concentration of the co-solvent increases reaching a constant value except for ethyl acetate where it was found to drop. Investigating flux and salt rejection by the formulated membranes showed that higher flux was attained when acetone was used as a co-solvent followed by diethyl ether then ethyl acetate. However, the highest salt rejection was achieved with diethyl ether.

## 1. Introduction

Membranes are widely used in many industrial applications such as desalination and water treatment [1,2,3,4]. Different types of membranes were developed for oil-water separation [5,6,7,8], industrial waste water treatment [9,10], organic matter removal from wastewater [11,12] and municipal waste water treatment [13,14,15,16].

A persistent problem associated with the application of membrane processes is membrane fouling defined as the reduction in the flux through the membrane due to the blocking of its pores or constriction due to the deposition of small particles [17,18]. To minimize this problem researchers have worked on finding new ways to manufacture membranes in which fouling problem is minimized yet keeping high salt rejection. Some example of these techniques are those related to the incorporation of nanoparticles during membrane formulation [19,20,21,22,23], the graft polymerization of ionic liquids with nanoparticles [24], the addition of co-solvent during the manufacturing process [25], the fabrication electrically conductive membranes based on carbon nanostructures [26], the preparation of permeable zeolite membranes [27,28,29], the interfacial polymerization with natural material [29,30] and the use of hydrophilic modifier [6,7,31,32,33,34,35,36].

A Unique technique to accomplish a high degree of water permeability is to develop reactivity of the monomer with additives in the aqueous phase. For instance, Hirose *et al.* stated that the addition of ether and alcohol in the water phase headed to greater water permeability and greater salt rejection [37,38,39]. Zhao *et al.* also stated that addition of hydrophilic additives such as anthranilic acid in the water phase headed to greater water permeability and higher salt rejection [40]. However, only a small number of articles have ever stated about the membranes produced with additives in the organic phase.

In our other study, we have achieved that acetone, ethyl acetate, and diethyl ether as co-solvents with hexane based solvent resulted in excellent salt rejection and high permeability [41]. The aim of this research is to investigate the effect of using other base solvents such as dodecane with the above mentioned cosolvents on the performance of reverse osmosis (RO) membranes. The structure of the produced membrane will be characterized by SEM, EDX, AFM and contact angle techniques. To the best of our knowledge, this is the first attempt to prepare and characterize thin film composite reverse osmosis (TFC RO) membrane in dodecane mixed with cosolvent.

## 2. Results and Discussion

### 2.1. SEM and EDX Analysis

**Figure 1:**
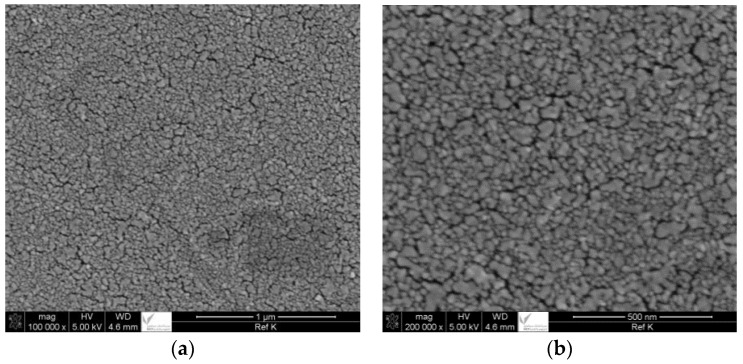
SEM images of PS membrane. (**a**) 100 k and (**b**) 200 k magnification.

Scanning electron microscope (SEM) of the polysulfone (PS) membrane surface at magnification of 100 k and 200 k is reported in Figure 1a,b respectively. It can be seen that the membrane surface is smooth.

**Figure 2:**
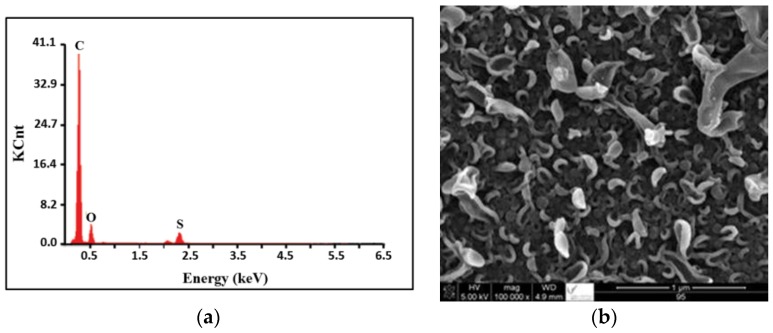
EDX spectrum (**a**) and (**b**) SEM image of TFC reference.

**Figure 3:**
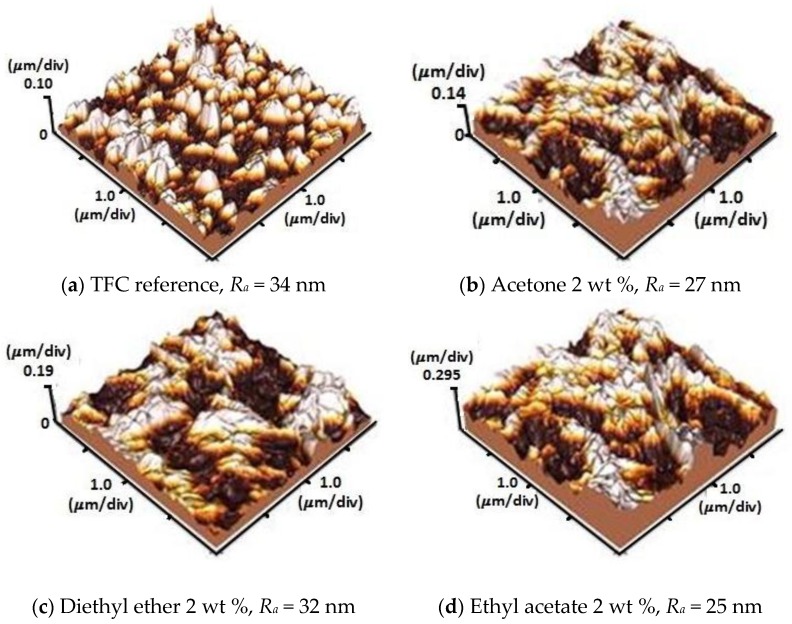
AFM images of formulated membranes with 2 wt % of co-solvents added (**a**) no co-solvent added; (**b**) acetone; (**c**) diethyl ether; and (**d**) ethyl acetate.

Images of the TFC reference (FT-30 membrane) by EDX and SEM portrayed in Figure 2a,b show that the surface roughness ranges between 25 and 34 nm. This is slightly rougher than that of the PS membrane (27–31 nm) [41] as indicated by the AFM images in Figure 3. The EDX spectrum shows that C, O, and S are the main elements exist in the TMC membrane.

**Figure 4:**
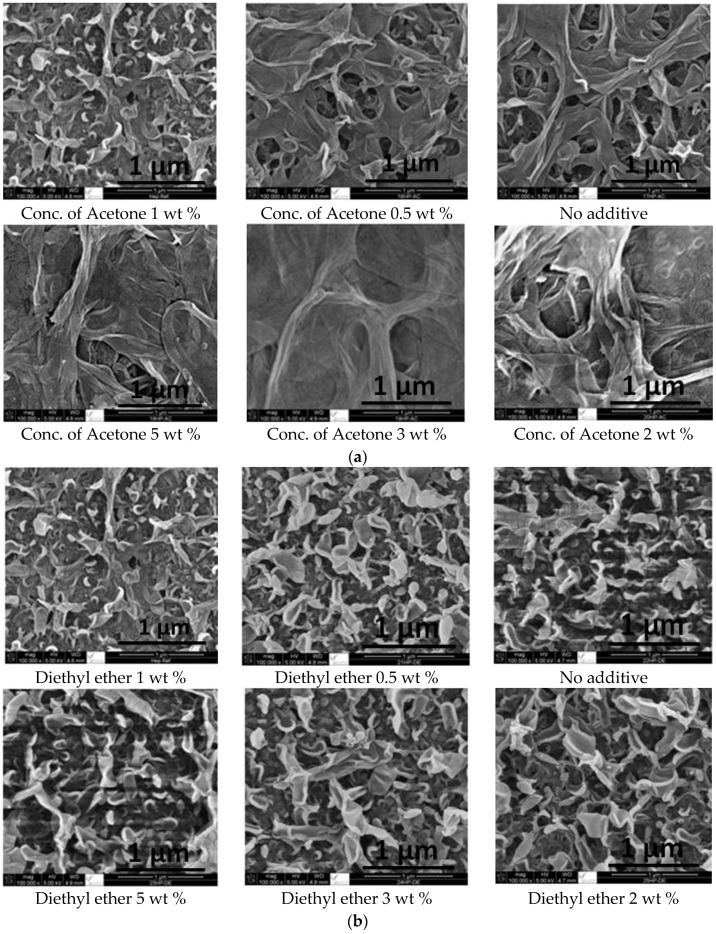

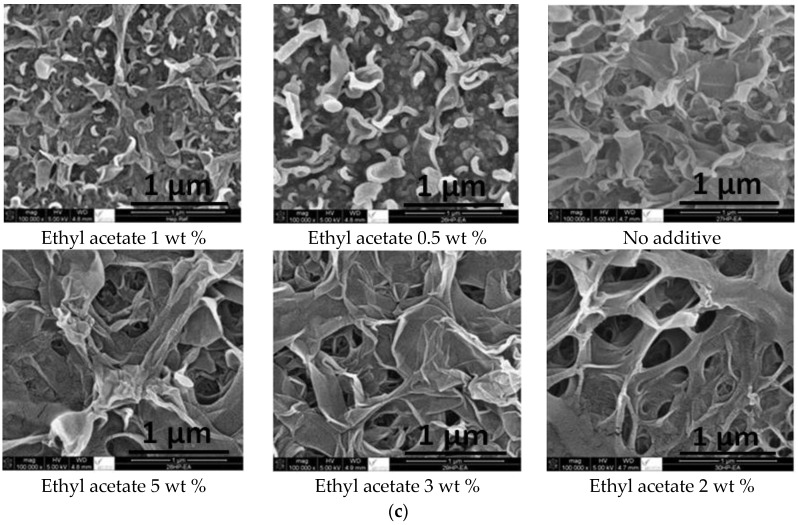
SEM images of the formulated membranes upon adding (**a**) acetone (**b**) diethyl ether (**c**) ethyl acetate.

The effect of adding co-solvents on the membrane morphology as shown has been investigated by SEM characterization (Figure 4a–c) for acetone, diethyl ether and ethyl acetate respectively. The images confirms the theory, *i.e.*, that the addition of a co-solvent introduces pores in the resulting membrane and pore size is proportional to co-solvent concentration [37,42,43,44]. The images of the SEM shows that pore size of membranes formulated with dodecane is larger than that formulated with hexane. This is possibly because dodecane has a larger molecular volume (0.2738 m^3^/kmol) than that of hexane (0.1406 m^3^/kmol). Therefore, molecular volume of the co-solvents plays a role in the pore size where the larger the molecular volume the larger the pore size [45].

**Figure 5:**
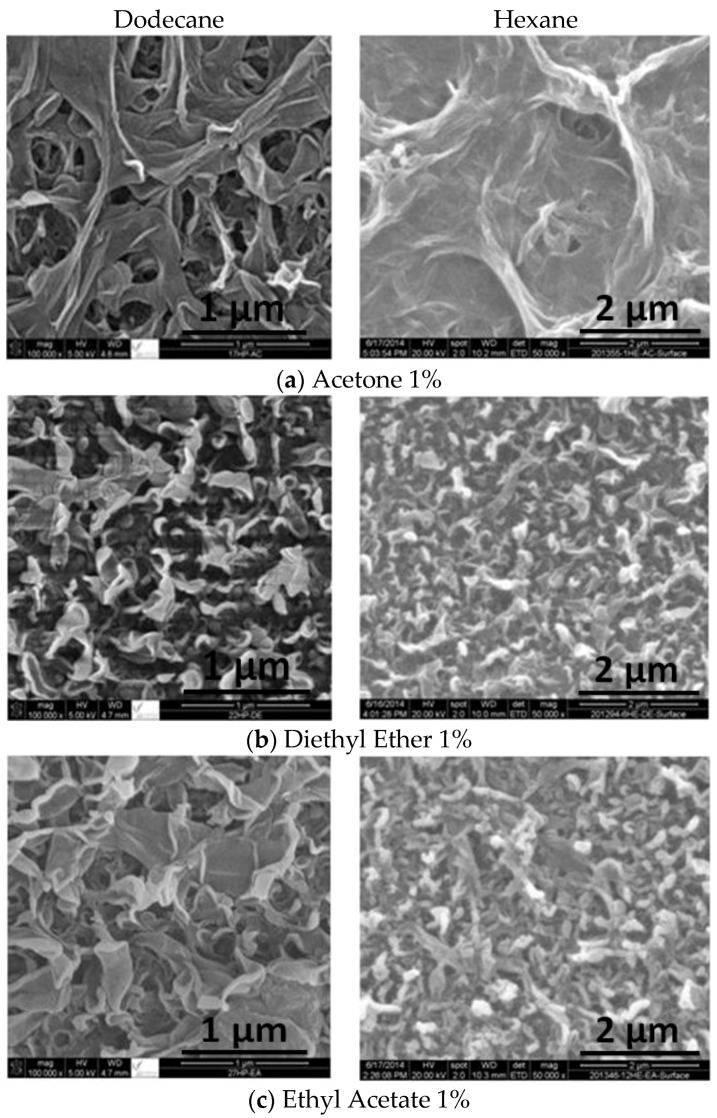
A comparison between dodecane (left) and hexane (right) on the morphology of the formulated membranes with 1% co-solvent concentration (**a**) acetone; (**b**) diethyl ether and (**c**) ethyl acetate.

The comparison of the surface morphology for the membranes prepared with two different solvent, *i.e.*, hexane as reported in [41] and dodecane (this study), at a co-solvents concentration (acetone, diethyl ether and ethyl acetate) of 1% is shown in Figure 5. The use of dodecane as solvent resulted in membrane with larger pore size (and hence the flux is expected to be larger as well) due to its reduced solubility (compared to hexane) with each of the three polar co-solvents giving to de-mixing phenomena [45]. Flux measurements and salt rejection are reported below in the permeate flux and salt rejection section. A comparison between the results of hexane and dodecane is reported and discussed as well.

### 2.2. Contact Angle Measurements

**Figure 6:**
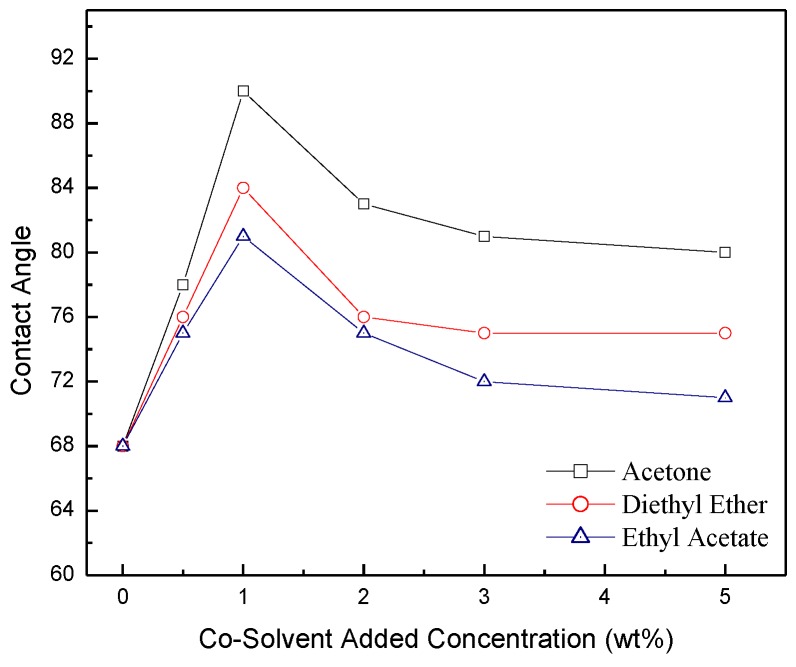
Contact angle as a function of co-solvent concentrations.

**Figure 7:**
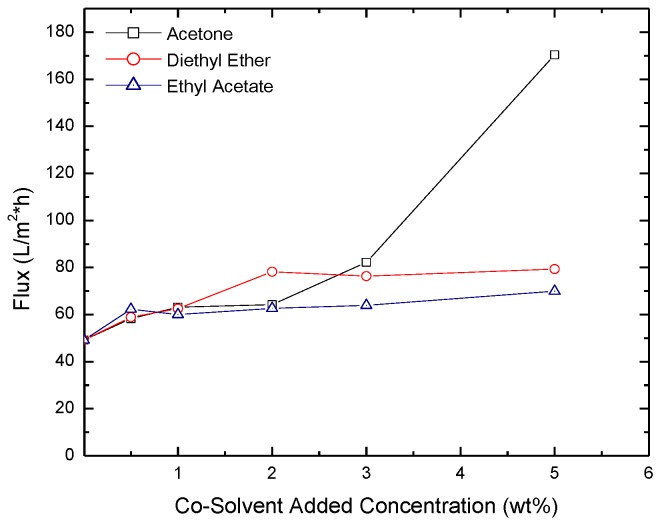
Flux of IPA through membranes formulated with acetone, diethyl ether and ethyl acetate added as co-solvents.

Measured contact angles for the co-solvents added as a function of their concentration are shown in Figure 6. It can be seen that acetone gives the highest contact angle followed by diethyl ether and ethyl acetate. For all measured membranes, the contact angle is < 90° indicating that they possess a hydrophilic property and hence they are less susceptible to fouling [46]. Figure 7 also shows that the contact angle for the three co-solvents tested goes through maxima at 1% co-solvent concentration. A similar trend was obtained for hexane with smaller contact angle at 1% co-solvent concentration. However, at larger and smaller co-solvent concentrations, the contact angle for dodecane solvent is smaller [41].

### 2.3. Permeate Flux

**Figure 8:**
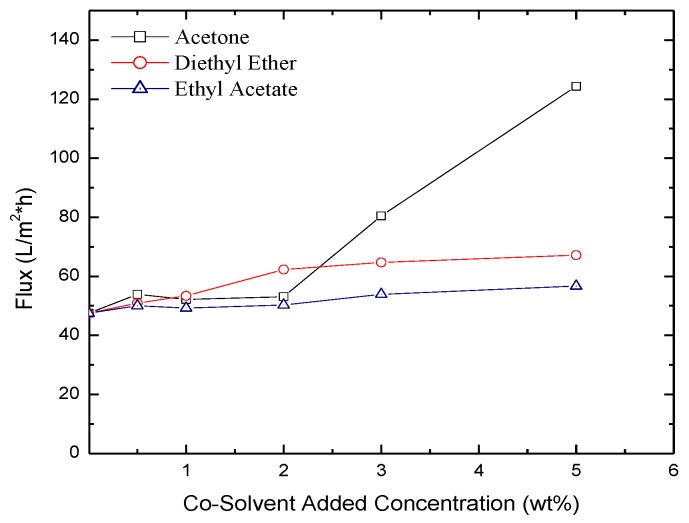
Flux of NaCl through membranes formulated with acetone, diethyl ether and ethyl acetate added as co-solvents.

**Figure 9:**
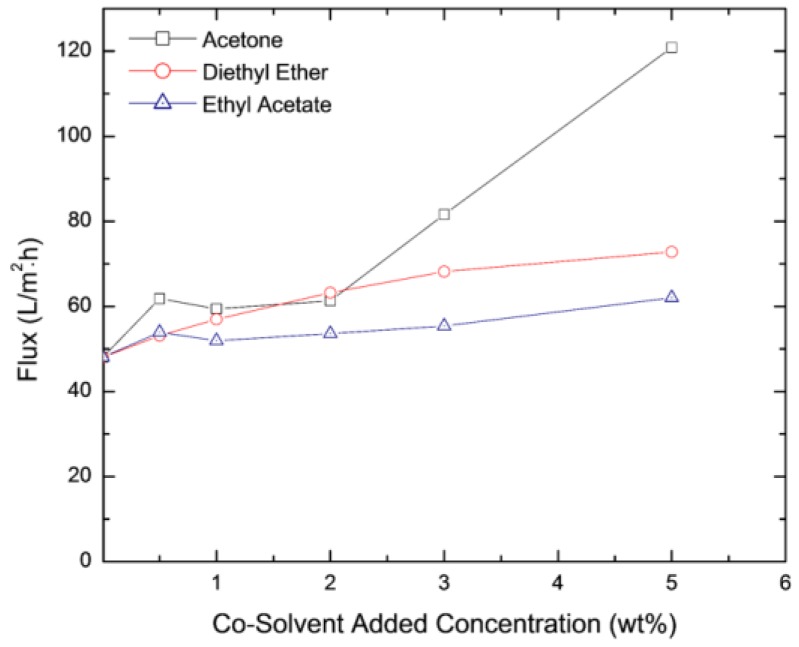
Flux of MgCl_2_ through membranes formulated with acetone, diethyl ether and ethyl acetate added as co-solvents.

Measured permeate flux through the membranes as a function of the concentration of the co-solvent added is reported in Figure 7, Figure 8 and Figure 9 for isopropyl alcohol (IPA), NaCl and MgCl_2_ solutions at a transmembrane pressure of 1.6 × 10^6^ Pa. The figures show that presence of the co-solvent increases the flux by ~25%. Except for acetone at concentration >3%, low effect of co-solvent concentration on flux was observed. From the SEM images above, Figure 5 shows that a large pore size membranes are obtained for acetone concentration >3% explaining the fast increase in the flux. The comparison of the fluxes with those obtained for hexane solvent shows that dodecane based membranes yield higher flux for all the salts tested [41]. This is because formulated membranes using dodecane as a cosolvent have larger pore size compared to those formulated using hexane as a co solvent. A comparison between the fluxes through the two types of membranes for the IPA, MgCl_2,_ and NaCl is shown in Table 1.

**Figure 10:**
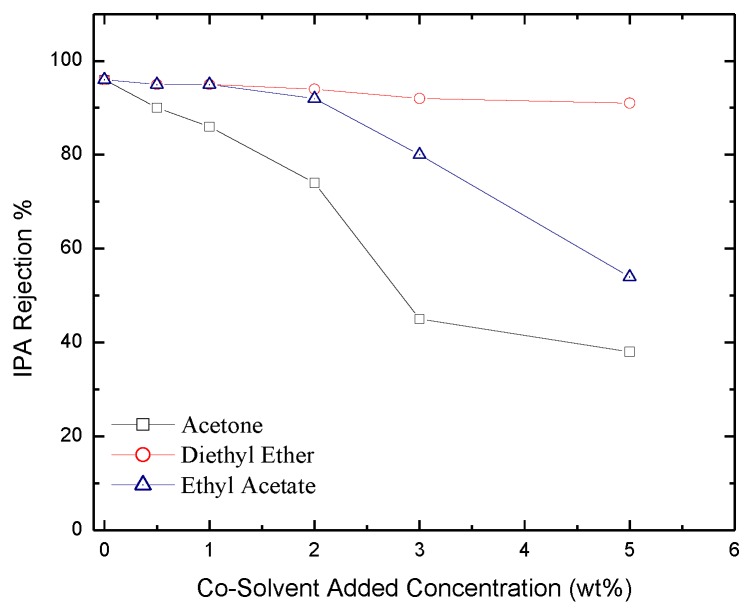
Salt rejection of IPA through membranes formulated with acetone, diethyl ether, and ethyl acetate added as co-solvents.

**Figure 11:**
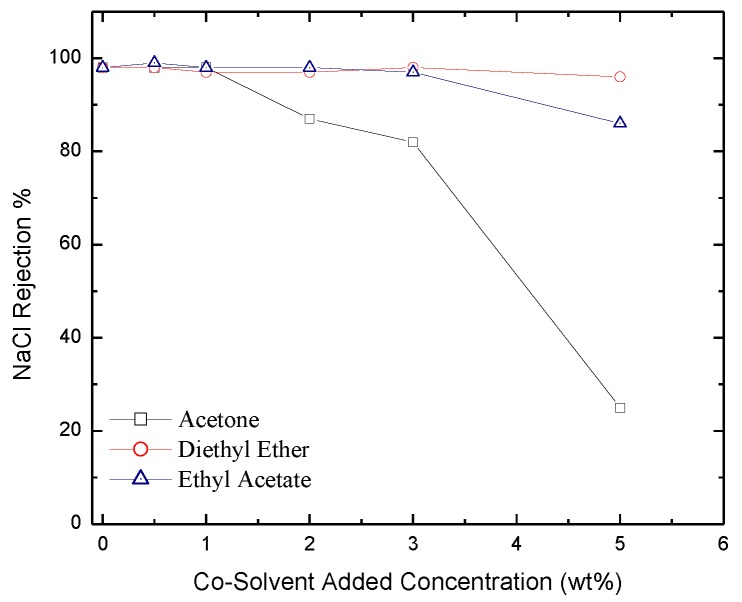
Experimentally measured salt rejection of NaCl through membranes formulated with acetone, diethyl ether, and ethyl acetate added as co-solvents.

**Figure 12:**
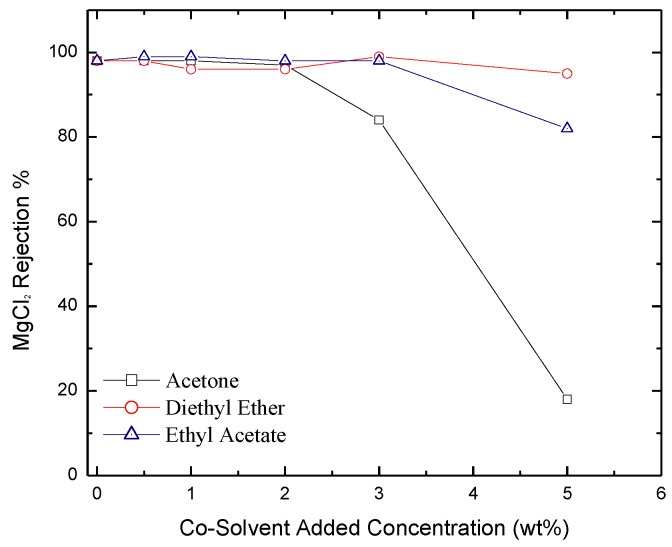
Salt rejection of MgCl_2_ through membranes formulated with acetone, diethyl ether, and ethyl acetate added as co-solvents.

*Salt Rejection*. Rejection of IPA, NaCl and MgCl_2_ salts as a function of co-solvent concentration is reported in Figure 10, Figure 11 and Figure 12 respectively. The figures show that salt rejection follows the order: diethyl ether > ethyl acetate > acetone. This is not surprising since acetone yields the highest pore size followed by ethyl acetate then diethyl ether (Figure 5). The figures also show that co-solvent concentration adversely affects salt rejection due to the formation of larger pore size. The comparison of these results with those obtained with hexane based membranes shows that dodecane based membranes provide larger salt rejection [41]. A comparison between the values of salt rejection through the two types of membranes for the IPA, MgCl_2_, and NaCl is shown in Table 1.

## 3. Experimental Section

### 3.1. Materials

All of the chemicals and reagents used in this study were purchased at the highest purity grades available.

### 3.2. Methods

#### Preparation of Modified Polyamide Membranes

Polyamide nanocomposite membranes were prepared on an ultrafiltration polysulphone (PS-20, Sepro, Oceanside, CA, USA) The microporous polysulphone supporting film with molecular weight cut-off (MWCO) of 20 kDa and Water Permeate Flux of 1000 LMH/bar, (Sepro, Oceanside, CA, USA) has been used as base for polymerization of 1,3-phenylenediamine (MPD, >99%, Sigma, St. Louis, MO, USA) with 1,3,5-benzenetricarbonyl trichloride (TMC, >98%, Sigma, St. Louis, MO, USA). The polyamide TFC membrane was produced by immersing PS-20 in an aqueous solution of 2% MPD for 2 min (the excess MPD solution was removed by rubber roller). The membrane is then immersed in 0.1% TMC/Dodecane solution (99%, Sigma) for 1 min with the required co-solvent. It is then rinsed with 0.2% Na_2_CO_3_ (>99%, Scharlau, Barcelona, Spain), washed with DI water, and finally stored in a refrigerator ≈4 °C in DI water until use. Acetone (>99%, Sigma, St. Louis, MO, USA), ethyl acetate (>99%, Sigma, St. Louis, MO, USA), and diethyl ether (>99%, Sigma, St. Louis, MO, USA) were selected as co-solvents with mass fraction of 0.5 wt %, 1 wt %, 2 wt %, 3 wt % and 5 wt %. The fabricated membranes were characterized by SEM, EDX, AFM and contact angle, (see next section for their specifications) to evaluate its performance with respect to salt rejection ability and water flux.

### 3.3. Characterization and Instrumentation

The following instrumentation techniques were used to characterize the developed membranes:

Scanning electron microscope (SEM) and energy-dispersive X-ray spectroscope (EDX). The morphology and microstructure of the as-synthesized TFC membranes were examined by scanning electron microscope (SEM, FEI Nova-Nano SEM-600, Eindhoven, The Netherlands). Quantitative analysis of the membranes was performed by energy-dispersive X-ray spectroscope (EDX).

Atomic force microscopy (AFM). Analysis of the surface morphology and roughness of the prepared membranes was performed by atomic force microscopy (AFM), using a Nan surf scanning probe-optical microscope (Bruker Corporation, Bremen, Germany). Small squares of the prepared membranes (approximately 1 cm^2^) were cut and glued onto glass substrates.

Contact angle. The contact angle was measured using a Ramé-Hart Model 250 Standard Goniometer/Tensiometer with DROP image Advanced software (Ramé-Hart Instrument Co., Succasunna, NJ, USA). A water droplet was placed on a dry flat homogeneous membrane surface and the contact angle between the water and membrane was measured until no further change was observed. The average contact angle for distilled water was determined in a series of eight measurements for each of the different membrane surfaces.

**Figure 13:**
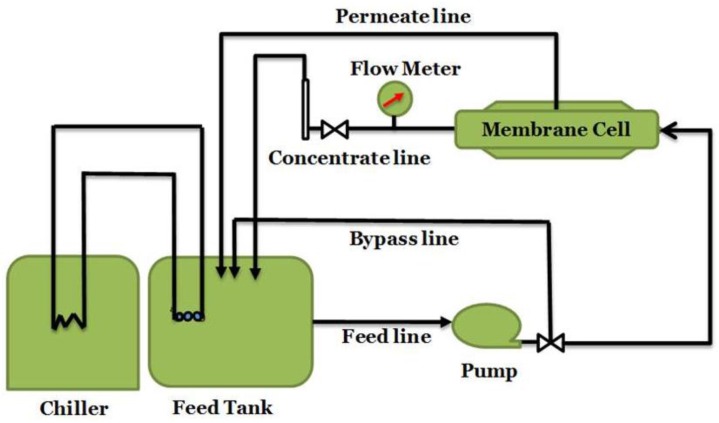
Schematic illustration of forward cross flow filtration system.

Cross-flow (flux and salt rejection). The performance of the prepared membranes was analyzed through a cross-flow system (CF042SS316 Cell, Sterlitech Corp., Kent, WA, USA). The active membrane area in this system was 42 cm^2^. The feed temperature was 25 °C with the pH adjusted to 6.5 ± 0.5, NaCl concentration of 2000 ppm and a flow rate of 3.8 L/min. The filtration was carried out at a transmembrane pressure of 1.6 × 10^6^ Pa. All measurements of the water flux and salt rejection were measured after 30 min of water filtration experiments to ensure that the system was at steady state. A schematic diagram of the cross-flow filtration system is shown in Figure 13.

The flux was calculated from the following equation [15]

(1) $$J=\;\frac{V_{p}}{A\;\times \;t}$$

where *J* is the water flux (L·m^−2^·h^−1^), *V_p_* is the permeate volume (L), *A* is the membrane area (m^2^) and *t* is the treatment time (h). Salt rejection (R) was calculated using the following equation:

(2) $$R=\;\left(1-\;\frac{C_{p}}{C_{f}}\right)\;\times 100$$

where *C_p_* and *C_f_* are the salt concentrations in the permeate and feed, respectively.

## 4. Conclusions

Highly efficient polyamide TFC RO membranes based on dodecane mixed with acetone, diethyl ether, or ethyl acetate as co-solvent were formulated and evaluated. The results show that addition of co-solvent resulted in membranes that have large pore size, low thickness, and high contact angle. At a given concentration, the following (decreasing) trend in terms of pore size and flux for the membranes prepared with the three co-solvents has been observed: acetone > diethyl ether > ethyl acetate. However, the highest IPA, NaCl, and MgCl_2_ rejections were attained when diethyl ether was used as co-solvent. The contact angle increases as the concentration of co-solvent increases and reaches a plateau, except for ethyl acetate, for which it was found to decrease. The contact angle was largest when acetone as a co-solvent was used. The experimental results showed that dodecane based membranes have better performance compared to hexane based membranes.

## Figures and Tables

**Table 1:** A comparison between the performance of hexane and dodecane based membranes. Data for hexane are obtained from Al-Hobaib *et al.* [41].

**Isopropanol (IPA)**												
(wt %)	**Co-Solvents Type**											
	**Acetone**				**Diethyl Ether**				**Ethyl Acetate**			
	***n*-Hexane**		***n*-Dodecane**		***n*-Hexane**		***n*-Dodecane**		***n*-Hexane**		***n*-Dodecane**	
	***F***	***S***	***F***	***S***	***F***	***S***	***F***	***S***	***F***	***S***	***F***	***S***
0	26	95	49	96	26	95	49	96	27	95	49	96
0.5	47	88	59	90	41	95	59	95	37	95	63	95
1	51	83	64	86	46	93	63	95	40	92	62	95
2	66	70	65	74	53	91	79	94	44	91	63	92
3	76	42	83	45	54	91	77	92	48	80	64	80
**NaCl**												
(wt %)	**Co-Solvents Type**											
	**Acetone**				**Diethyl Ether**				**Ethyl Acetate**			
	***n*-Hexane**		***n*-Dodecane**		***n*-Hexane**		***n*-Dodecane**		***n*-Hexane**		***n*-Dodecane**	
	***F***	***S***	***F***	***S***	***F***	***S***	***F***	***S***	***F***	***S***	***F***	***S***
0	26	99	47	99	26	99	47	98	27	98	47	98
0.5	39	96	53	99	36	96	51	98	34	98	50	99
1	41	93	53	98	41	96	54	98	38	97	50	98
2	56	83	54	97	45	92	63	87	42	97	51	98
3	67	37	81	97	42	80	65	82	44	98	54	97
**MgCl_2_**												
(wt %)	**Co-Solvents Type**											
	**Acetone**				**Diethyl Ether**				**Ethyl Acetate**			
	***n*-Hexane**		***n*-Dodecane**		***n*-Hexane**		***n*-Dodecane**		***n*-Hexane**		***n*-Dodecane**	
	***F***	***S***	***F***	***S***	***F***	***S***	***F***	***S***	***F***	***S***	***F***	***S***
0	27	99	48	98	27	99	48	98	27	99	48	98
0.5	41	97	62	98	42	98	54	98	35	95	54	99
1	45	94	61	98	40	97	57	96	39	95	53	99
2	56	88	63	97	47	96	64	96	44	92	54	98
3	74	26	82	84	44	97	69	99	45	77	56	98

*F* = Flux (L/m^2^·h) and *S* = % Salt rejection.
